# Cost-effectiveness of chemotherapy in advanced and recurrent endometrial cancer

**DOI:** 10.1016/j.gore.2025.101761

**Published:** 2025-05-05

**Authors:** Katsuaki Inami

**Affiliations:** Department of Obstetrics and Gynecology, Fujinomiya City General Hospital, 3-1 Nishiki-cho, Fujinomiya, Shizuoka, Japan

**Keywords:** Endometrial cancer, Cost-effectiveness, Chemotherapy, Immunotherapy, Incremental cost-effectiveness ratio, Advanced cancer, Recurrent cancer, Health economics

## Abstract

**Objective:**

To review the cost-effectiveness of chemotherapy and immunotherapy-based regimens for advanced and recurrent endometrial cancer, focusing on incremental cost-effectiveness ratios (ICERs).

**Methods:**

We conducted a literature review of peer-reviewed studies (2021–2025) evaluating immune checkpoint inhibitors (ICIs) combined with chemotherapy or targeted agents versus standard chemotherapy in advanced/recurrent endometrial cancer. Key outcomes (cost per QALY or life-year gained, willingness-to-pay [WTP] thresholds) and conclusions were extracted from nine studies.

**Results:**

Adding ICIs to first-line chemotherapy improved survival, especially in mismatch repair-deficient (dMMR) tumors. In dMMR disease, pembrolizumab or dostarlimab plus chemotherapy yielded ICERs of $41,000–$60,000/QALY, considered cost-effective at a $150,000/QALY threshold, but not at $100,000/QALY without price reductions. In mismatch repair-proficient (pMMR) patients, first-line ICI combinations showed smaller QALY gains and higher ICERs ($90,000–$176,000/QALY), often exceeding accepted thresholds. The durvalumab + olaparib combination was not cost-effective in any subgroup (ICERs >$200,000/QALY). In recurrent pMMR disease, pembrolizumab + lenvatinib was not cost-effective in U.S. or Chinese settings unless drug costs declined by 8–50 %. In recurrent dMMR cancer, dostarlimab improved outcomes but had an ICER of $332,000/QALY, making it economically unjustified at current prices.

**Conclusions:**

ICIs offer clinical benefit in advanced endometrial cancer, particularly in dMMR tumors. First-line ICI + chemotherapy appears cost-effective for dMMR at U.S. WTP levels. However, use in pMMR or second-line settings may require drug price reductions or biomarker-based selection to be economically viable.

## Highlights

• Cost-effectiveness of immune checkpoint inhibitors for advanced endometrial cancer is reviewed.
• Pembrolizumab or dostarlimab plus chemotherapy is cost-effective for dMMR tumors.
• Cost-effectiveness in pMMR disease is limited due to modest survival gains and high drug costs.
• Use of ICIs in second-line settings requires price reductions to be economically viable.
• Biomarker-based patient selection may improve value of immunotherapy in clinical practice.

## 1 Introduction

Advanced and recurrent endometrial cancer carry a poor prognosis and impose a growing health burden. Approximately 10–15 % of endometrial cancer patients present with advanced-stage disease, and up to 50 % of those treated with first-line platinum/taxane chemotherapy will experience recurrence (Huo et al., 2024). For decades, the standard systemic therapy for metastatic or recurrent endometrial carcinoma has been combination carboplatin and paclitaxel, which yields a median overall survival on the order of 2–3 years in advanced disease. Outcomes are particularly poor in the majority of tumors that are mismatch repair-proficient (pMMR) or microsatellite stable (MSS), as these tend to be less responsive to immunotherapy and have limited options after first-line chemotherapy failure. In contrast, the 15–30 % of endometrial cancers with mismatch repair deficiency (dMMR) or microsatellite instability-high (MSI-H) may respond dramatically to immune checkpoint inhibitors (ICIs). Over the past five years, several landmark trials have demonstrated improved survival by incorporating ICIs into the treatment of advanced endometrial cancer. For example, the phase III trials NRG-GY018 and RUBY showed that adding anti–PD-1 antibodies (pembrolizumab or dostarlimab) to first-line chemotherapy significantly prolonged progression-free survival, especially in dMMR patients, leading to regulatory approvals (Kim et al., 2023) of these combinations as new standards of care. Additionally, the combination of pembrolizumab with lenvatinib (an oral multikinase inhibitor) has shown efficacy in previously treated pMMR endometrial cancer (the KEYNOTE-775 trial), and ICIs such as dostarlimab have demonstrated high response rates as monotherapy in dMMR recurrent cases (Dioun et al., 2023).

While these novel immunotherapy-based regimens have improved clinical outcomes, they come with substantial costs. ICIs like pembrolizumab, dostarlimab, and durvalumab are expensive therapies, often exceeding $10,000 per dose, and targeted agents like lenvatinib and olaparib also add to the expense. This raises the critical question of cost-effectiveness: do the gains in survival and quality of life provided by these treatments justify their incremental costs relative to standard chemotherapy? Cost-effectiveness analysis (CEA) is a key tool in health economics to inform such decisions, typically using the incremental cost-effectiveness ratio (ICER)—the additional cost divided by additional effectiveness (e.g. cost per QALY or per LY gained) of a new strategy compared to an alternative. An ICER below a society’s willingness-to-pay (WTP) threshold (e.g. $100,000–$150,000 per QALY in the United States) suggests the new treatment provides acceptable “value for money,” whereas an ICER above the threshold indicates the therapy may not be economically justified (Neumann et al., 2014).

Multiple CEA studies have recently evaluated immunotherapy and combination regimens in advanced or recurrent endometrial cancer. This article systematically reviews the current evidence on the cost-effectiveness of these regimens, with an emphasis on balancing clinical effectiveness and economic value. We focus on nine key analyses published from 2021 through 2025, which examined: (1) ICIs (pembrolizumab or dostarlimab) added to first-line chemotherapy for advanced/recurrent disease; (2) the combination of ICIs with other agents (lenvatinib or olaparib) in various settings; and (3) ICI monotherapy in biomarker-selected recurrent disease. We summarize their findings on costs, QALYs, and ICERs, and discuss the implications for treatment selection and policy.

## 2 Methods

A literature search was conducted to identify economic evaluations of chemotherapy and immunotherapy regimens in advanced or recurrent endometrial cancer. We searched PubMed and major oncology conferences (through 2025) for cost-effectiveness analyses pertaining to endometrial cancer systemic therapies. Studies were included if they were peer-reviewed articles assessing the cost-effectiveness of chemotherapy and/or ICI-based treatments (including combination therapies) for advanced or recurrent endometrial cancer, using outcomes such as cost per QALY or cost per life-year. For regimens that included continued immunotherapy after initial chemotherapy (i.e., maintenance IO), the total modeled costs reflected the full duration of treatment, including the maintenance phase. This approach contrasts with chemotherapy-alone arms, which typically discontinued treatment upon disease response or stable disease. Nine relevant studies were identified, each evaluating a distinct regimen or comparison of interest: Ackroyd et al. 2021 (Ackroyd et al., 2021), Kim et al. 2023 (Kim et al., 2023), Huo et al. 2024 (pembrolizumab) (Huo et al., 2024), Huo et al. 2024 (dostarlimab) (Huo et al., 2024), Liu et al. 2025 (Liu et al., 2025), Zhang et al. 2025 (Zhang et al., 2025), Diggs et al. 2023 (Diggs et al., 2023), Sun et al. 2022 (Sun et al., 2022), and Dioun et al. 2023 (Dioun et al., 2023). Data were extracted on the treatment strategies compared, target patient population (e.g. dMMR or pMMR, line of therapy), analytic perspective, key results (costs, QALYs, ICERs), assumed WTP thresholds, and authors’ conclusions regarding cost-effectiveness. All costs are reported in 2020–2025 U.S. dollars (USD) for consistency, with foreign currency results converted to USD if necessary. Below, we present the results organized by line of therapy and patient population, and a comparative summary table (Table 1).

**Table 1:** Summary of cost-effectiveness results for immunotherapy-containing regimens in advanced or recurrent endometrial cancer.

Study (Year)	Population / Setting	Treatment comparison	ICER (cost/QALY)	WTP threshold	Conclusion on cost-effectiveness
Ackroyd et al, 2021 (Ackroyd et al., 2021)	First-line advanced/recurrent EC; MSS and MSI-H cohorts analyzed separately	Pembro + lenvatinib vs carboplatin/paclitaxel (chemo)	MSS: Dominated; MSI-H: $2,849,882/QALY	$150,000/QALY (USA)	Not cost-effective (MSS: inferior to chemo; MSI-H: high ICER, ∼85 % price reduction needed)
Kim et al, 2023 (Kim et al., 2023)[a][b]	First-line primary advanced dMMR EC	Chemo (TC) vs TC + pembrolizumab vs TC + dostarlimab	Pembrolizumab: $377,718/QALY; Dostarlimab: Dominated (∼$401 k/QALY)	$100,000/QALY (USA)	Not cost-effective; pembrolizumab needs ∼ 61 % cost reduction to meet $100 k/QALY
Huo et al, 2024 (Huo et al., 2024)	First-line advanced/recurrent EC; dMMR and pMMR cohorts	Chemo vs chemo + pembrolizumab	dMMR: $41,305/QALY; pMMR: $90,285/QALY	$150,000/QALY (USA)	Cost-effective in both (high value in dMMR, borderline in pMMR)
Huo et al, 2024 (Huo et al., 2024)	First-line primary advanced/recurrent EC; dMMR and pMMR	Chemo vs chemo + dostarlimab	dMMR: $60,349/QALY; pMMR: $175,788/QALY	$150,000/QALY (USA)	Cost-effective in dMMR; not in pMMR (∼15 % price cut needed)
Liu et al, 2025 (Liu et al., 2025)	First-line advanced/metastatic EC (∼74 % pMMR)	Chemo vs chemo + pembrolizumab vs chemo + dostarlimab	pMMR: Pembro $974 k/QALY, Dostarlimab $234 k/QALY; dMMR: Pembro $266 k, Dostarlimab $135 k	$150,000/QALY (USA)	Dostarlimab preferred for dMMR; neither cost-effective for pMMR
Zhang et al, 2025 (Zhang et al., 2025)	First-line advanced/recurrent EC; dMMR and pMMR (DUO-E trial)	Chemo vs chemo + durvalumab ± olaparib	dMMR: $477 k–$584 k/QALY; pMMR: $220 k–$352 k/QALY	$150,000/QALY (USA)	Not cost-effective (all ICERs exceed threshold)
Diggs et al, 2023 (Diggs et al., 2023)	Second-line recurrent pMMR EC (USA)	Chemo vs pembrolizumab + lenvatinib	$164,493/QALY	$150,000/QALY (USA)	Not cost-effective; ∼8% drug price reduction needed
Sun et al, 2022 (Sun et al., 2022)	Second-line pMMR EC (China)	Chemo vs pembrolizumab + lenvatinib	$70,962/QALY	∼$37,700/QALY (China)	Not cost-effective; large price cut needed
Dioun et al, 2023 (Dioun et al., 2023)	Second-line recurrent dMMR EC	PLD vs dostarlimab vs pembrolizumab	Dostarlimab: $331,913/QALY; Pembro: dominated	$100,000/QALY (USA)	Not cost-effective; ∼50 % price cut needed

a This study used a $100,000 per QALY willingness-to-pay threshold, whereas most others used $150,000/QALY.

b In Kim et al. (2023), dostarlimab + chemotherapy was dominated by pembrolizumab + chemotherapy, meaning it was both more costly and less effective. As such, further incremental analysis was performed using pembrolizumab + chemotherapy versus chemotherapy alone. The ∼ 61 % price reduction refers to pembrolizumab’s cost required to meet a $100,000/QALY threshold.

## 3 Cost-effectiveness of first-line therapy regimens

### 3.1 ICIs added to first-line chemotherapy in advanced endometrial cancer

**dMMR (MSI-H) population:** Patients with primary advanced or recurrent endometrial cancer whose tumors are dMMR derive the largest survival benefit from adding ICIs to upfront chemotherapy. In a cost-effectiveness analysis informed by the NRG-GY018 (pembrolizumab) and RUBY (dostarlimab) trial results, Kim et al. found that adding pembrolizumab to first-line carboplatin/paclitaxel (TC) in dMMR disease increased quality-adjusted survival but was not cost-effective at conventional thresholds: the ICER for pembrolizumab + chemo versus chemo alone was approximately $377,700 per QALY, far above a $100,000/QALY threshold (Kim et al., 2023). Dostarlimab + chemo was even less favorable, being ruled “absolutely dominated” (more costly and less effective than the pembrolizumab regimen) with an implied ICER around $402,000/QALY (Kim et al., 2023). In this analysis, dostarlimab + chemotherapy was found to be dominated by pembrolizumab + chemotherapy—being more expensive and less effective—and was therefore excluded from further cost-effectiveness comparisons. The focus shifted to evaluating whether pembrolizumab could meet WTP thresholds if drug prices were reduced. Kim et al. concluded that, at current drug prices, first-line ICI combinations in dMMR patients would not be cost-effective without substantial price reductions – they estimated pembrolizumab’s cost per 200 mg dose would need to decrease by ∼ 61 % (to ∼$4,361) for the regimen to meet a $100,000/QALY threshold (Kim et al., 2023).

In contrast, analyses using a higher WTP threshold and longer time horizon have reported more optimistic results for this population. Huo et al. evaluated pembrolizumab with chemo versus chemo alone in advanced dMMR endometrial cancer from a U.S. payer perspective, using a lifetime horizon and a $150,000/QALY threshold (Huo et al., 2024). Willingness-to-pay (WTP) thresholds are typically based on a country’s economic context, including per-capita health expenditures, societal preferences, and policy norms. For example, the United States commonly applies thresholds ranging from $100,000 to $150,000 per QALY, while countries like the United Kingdom—through agencies such as NICE—use lower thresholds, approximately £20,000 to £30,000 per QALY. These differences mean that a treatment deemed cost-effective in one country may not be considered so in another, highlighting the importance of contextual interpretation. While most of the reviewed U.S.-based studies adopted a $150,000 per QALY threshold, Kim et al. (2023) applied a more conservative $100,000 per QALY threshold. This reflects differences in modeling assumptions and institutional perspectives regarding acceptable cost-effectiveness levels within the same national context. They found that pembrolizumab + chemotherapy provided an incremental 4.05 QALYs per patient at an added cost of $167,224, yielding an ICER of only $41,305 per QALY (Huo et al., 2024). This is well below $150,000 and even under $50,000 per QALY, indicating high cost-effectiveness in dMMR patients under those assumptions. The model predicted substantial gains in life expectancy with the immunotherapy combination (reflecting the dramatic survival improvements seen in dMMR tumors), and probabilistic sensitivity analysis showed a 100 % probability that pembrolizumab was cost-effective at the $150,000/QALY threshold. Similarly, Huo et al. analyzed dostarlimab + chemo in the same setting (based on RUBY trial data) and found an ICER of $60,349 per QALY gained for dMMR patients (Huo et al., 2024). The dostarlimab regimen added an estimated 5.48 QALYs at an additional cost of about $331,000 versus chemo alone, thus remaining below $150,000/QALY (indeed, even below $100,000/QALY) for dMMR disease (Huo et al., 2024). Their simulation also indicated a 100 % chance of cost-effectiveness at the $150,000 threshold for dostarlimab in dMMR patients. In summary, using U.S. cost assumptions, ICI + chemotherapy in dMMR advanced endometrial cancer appears to be an economically reasonable strategy (ICER ∼$41–60 K/QALY) when evaluated over a lifetime horizon, whereas under more conservative assumptions (shorter horizon, $100 K threshold) it would not meet typical cost-effectiveness cutoffs (Kim et al., 2023). All analyses agree that the large drug costs are a concern, and results are highly sensitive to ICI pricing – even the favorable Huo models noted drug cost as a top driver of the ICER results.

**pMMR (MSS) population:** For the majority of advanced endometrial cancers that are pMMR, adding an ICI to first-line chemotherapy yields more modest clinical benefits, and cost-effectiveness is correspondingly less favorable. In Kim et al.’s model (restricted to dMMR tumors), the pMMR group was not analyzed for ICI benefit (since GY018 and RUBY showed minimal advantage in pMMR). However, Huo et al. assessed pembrolizumab’s value in pMMR disease using the subgroup data from NRG-GY018. They found an ICER of $90,285/QALY for pembrolizumab + chemo versus chemo alone in pMMR patients (Huo et al., 2024), based on a gain of 0.93 QALYs at an added cost of ∼$83,600. This ICER is below $150,000, suggesting borderline cost-effectiveness in the U.S. context, though it is slightly above a $100,000 threshold. In contrast, dostarlimab + chemo in pMMR patients was not cost-effective in Huo’s analysis: the ICER was $175,788/QALY, exceeding $150,000 (Huo et al., 2024). The difference reflects that in the RUBY trial, the pMMR subset derived smaller incremental QALY gains from dostarlimab. Indeed, Huo et al. determined that a dostarlimab price discount of about 15 % (reducing cost to reach an ICER of $150,000/QALY) would be required for the pMMR subgroup (Huo et al., 2024). The probability of cost-effectiveness for dostarlimab in pMMR patients was essentially near 0 % at WTP $150 K (Huo et al., 2024), versus near 100 % in dMMR. Similarly, Liu et al. compared pembrolizumab + chemo and dostarlimab + chemo head-to-head in a modeled pMMR cohort: neither regimen was cost-effective vs chemotherapy at $150,000/QALY, with ICERs of approximately $974,000/QALY for pembrolizumab and $234,500/QALY for dostarlimab (each vs chemo) (Liu et al., 2025). When comparing the two ICI regimens directly in pMMR disease, dostarlimab + chemo dominated pembrolizumab + chemo (providing more QALYs at slightly lower cost), with an ICER of $86,671/QALY for dostarlimab vs pembrolizumab (Liu et al., 2025). This suggests that if an ICI were to be used in pMMR patients, the dostarlimab regimen might offer better relative value, but overall these ICERs are high. Taken together, current evidence indicates that routine addition of immunotherapy to first-line chemo in pMMR advanced endometrial cancer may not be cost-effective unless drug prices decrease or a higher WTP threshold (>$150 K) is accepted. Pembrolizumab + chemo yielded a borderline ICER ($90 K/QALY) in one U.S. model (Huo et al., 2024), but dostarlimab + chemo was not economically justified for pMMR without discounts (Huo et al., 2024).

### 3.2 Durvalumab (± olaparib) with first-line chemotherapy

The DUO-E trial investigated another strategy: adding the PD-L1 inhibitor durvalumab to first-line chemo, with or without maintenance olaparib (a PARP inhibitor), in advanced endometrial cancer. Zhang et al. performed a cost-effectiveness analysis of these regimens (durvalumab + chemo followed by durvalumab maintenance [“DCT”], and durvalumab + chemo with maintenance durvalumab + olaparib [“DOCT”]) versus chemotherapy alone, stratifying by MMR status (Zhang et al., 2025). In dMMR patients, both durvalumab-containing strategies were associated with large cost increases and yielded ICERs far above typical thresholds: approximately $476,946/QALY for DCT and $584,141/QALY for DOCT (each vs chemo) (Zhang et al., 2025). The durvalumab arms actually had higher total costs than chemo even in dMMR (the model estimated chemo-alone cost at ∼$1.5 million per patient with chemo vs ∼$1.2 million with DOCT in dMMR, reflecting higher costs during prolonged survival). In pMMR disease, the ICERs were also not favorable: ∼$219,600/QALY for DCT and ∼$351,800/QALY for DOCT, each vs chemo (Zhang et al., 2025). One of the main reasons for the variation in ICERs across different chemo-immunotherapy combinations lies in the differences in both cost and incremental clinical benefit. For instance, the durvalumab-based regimens—especially those incorporating olaparib maintenance—incurred substantially higher total costs than regimens based on pembrolizumab. At the same time, the QALY gains achieved with durvalumab combinations were relatively modest in comparison. This led to significantly higher ICERs for durvalumab-containing strategies, often exceeding $250,000 per QALY, whereas pembrolizumab-based combinations demonstrated greater survival benefits at lower additional costs. These differences highlight how small variations in efficacy and pricing can lead to large differences in cost-effectiveness outcomes. When considering the overall trial population, neither regimen was cost-effective; DOCT had an ICER of ∼$269,000/QALY and DCT ∼$416,000/QALY vs chemo (Zhang et al., 2025). Zhang et al. concluded that at a $150,000/QALY WTP threshold, adding durvalumab (with or without olaparib) cannot be justified economically for advanced endometrial cancer (Zhang et al., 2025). The addition of olaparib, which further increases cost, was particularly unfavorable in cost-effectiveness terms, providing minimal additional QALY gain for a very high ICER. Thus, despite promising biological rationale, the durvalumab ± olaparib strategy does not appear to offer good value at current drug prices and efficacy levels.

### 3.3 Pembrolizumab + lenvatinib vs chemotherapy as first-line therapy

An alternative approach examined by Ackroyd et al. was to use the combination of pembrolizumab and lenvatinib (P + L) in the first-line setting instead of the standard carboplatin/paclitaxel chemotherapy. Pembrolizumab + lenvatinib is an approved regimen for second-line treatment of pMMR endometrial cancer, but Ackroyd et al. modeled its hypothetical use as initial therapy (replacing chemo) in advanced/recurrent disease, with separate cohorts of MSS (pMMR) and MSI-H (dMMR) tumors (Ackroyd et al., 2021). The findings strongly favored standard chemotherapy from both a clinical and economic perspective. In the MSS group, first-line P + L was actually less effective than chemo – it produced 0.28 fewer QALYs per patient and cost an additional $212,670, meaning P + L was strictly dominated by chemotherapy (Ackroyd et al., 2021). This reflects that carboplatin/paclitaxel is an effective initial regimen in MSS cancers, and delaying or replacing it with P + L led to worse survival outcomes in the model. In the MSI-H cohort, pembrolizumab + lenvatinib did improve QALYs by 0.11 compared to chemo, but at a cost increase of $313,487, resulting in an ICER of approximately $2.85 million per QALY (Ackroyd et al., 2021). This astronomically exceeds any plausible threshold. Sensitivity analyses confirmed that drug costs were the primary driver: the price of P + L would need to drop by ∼ 85 % (to roughly $2,800 per cycle) to reach a $150,000/QALY threshold (Ackroyd et al., 2021). The authors concluded that first-line P + L is not cost-effective for either MSS or MSI-H endometrial cancer at current prices, and in MSS disease it may even worsen patient outcomes relative to standard chemotherapy (Ackroyd et al., 2021). These results underscore that, at least in the absence of chemotherapy, the P + L regimen cannot economically (or perhaps even clinically) outperform the established first-line chemo doublet. This contrasts with the strategy of **adding** immunotherapy on top of chemo (as in GY018/RUBY), which does yield outcome benefits; thus, replacing chemo entirely with P + L upfront appears unjustified both in health and economic terms given present evidence.

### 3.4 Cost-effectiveness in the recurrent or second-line setting

#### 3.4.1 Pembrolizumab + lenvatinib for recurrent pMMR endometrial cancer

For patients with advanced endometrial cancer that has relapsed or progressed after first-line chemotherapy, the combination of pembrolizumab plus lenvatinib is an approved therapy for those with pMMR (MSS) tumors, based on improved survival seen in the phase III KEYNOTE-775 trial. Several analyses have evaluated the cost-effectiveness of P + L versus standard chemotherapy in this setting. In a U.S. study, Diggs et al. examined P + L versus chemotherapy (doxorubicin or weekly paclitaxel) in women with recurrent pMMR endometrial cancer after platinum therapy (Diggs et al., 2023). Over a 2-year horizon, P + L resulted in more patients alive at 2 years (approximately 36.7 % vs 10.9 %) and higher QALYs, but also much higher costs. The total cost per patient on P + L was about $193,590 compared to $66,693 on chemotherapy (Diggs et al., 2023). The resulting ICER for P + L was approximately $164,500 per QALY gained versus chemo (Diggs et al., 2023). Using a $150,000/QALY threshold, this ICER was slightly above the acceptable range (making chemotherapy the preferred cost-effective option) (Diggs et al., 2023). However, only a modest price reduction would be needed to tip the balance: the authors calculated that if the monthly cost of P + L were reduced by about $1,553 (around 7.8 % of the price), the ICER would fall below the threshold, making the regimen cost-effective (Diggs et al., 2023). In their conclusion, Diggs et al. noted that pembrolizumab plus lenvatinib is associated with a meaningful survival benefit but requires a small decrease in drug price (approximately 7 %–8%) to be considered cost-effective for recurrent pMMR endometrial cancer in the U.S. setting (Diggs et al., 2023).

From a Chinese health system perspective, where drug pricing and WTP thresholds differ, the cost-effectiveness of P + L appears less favorable. Sun et al. performed a CEA of P + L versus chemotherapy for previously treated pMMR endometrial cancer in China (Sun et al., 2022). Even though costs were lower in absolute terms, they found an ICER of approximately $70,962 per QALY for P + L versus chemo (Sun et al., 2022), exceeding the commonly used Chinese WTP threshold of about $37,700/QALY (Sun et al., 2022). In the base case, P + L provided 0.89 additional QALYs at an incremental cost of ∼$62,900 (Sun et al., 2022), which was not cost-effective in China. Sensitivity analyses confirmed the robustness of this finding, with drug costs and the number of P + L cycles being influential parameters. Sun et al. concluded that P + L is not cost-effective in the Chinese context unless the price is significantly reduced (Sun et al., 2022). In fact, given the ICER was nearly double the threshold, a price cut on the order of 40–50 % might be required to meet the criterion. These results highlight that while P + L is a clinically beneficial second-line option, its economic value is highly contingent on drug pricing and local WTP thresholds. In higher-income settings (with higher WTP), it may be close to cost-effective (needing minor price concessions), whereas in lower WTP settings like China, it is far from cost-effective at current prices.

#### 3.4.2 Immunotherapy monotherapy in recurrent dMMR endometrial cancer

Immune checkpoint blockade as monotherapy has become an important strategy for patients with dMMR recurrent or metastatic endometrial cancer, many of whom experience durable responses. Dostarlimab, for instance, received accelerated FDA approval for recurrent dMMR endometrial cancer after chemotherapy. Dioun et al. evaluated the cost-effectiveness of using dostarlimab in this scenario compared to other options (Dioun et al., 2023). Their analysis modeled a cohort of women with recurrent dMMR tumors after first-line chemo, comparing three strategies: treatment with dostarlimab, pembrolizumab, or pegylated liposomal doxorubicin (PLD) chemotherapy. The results showed that PLD (an active chemotherapy in this setting) was the least costly strategy (about $55,732 per patient), but also the least effective, while immunotherapy improved QALYs. Dostarlimab was associated with greater effectiveness than either PLD or pembrolizumab. Pembrolizumab was found to be “extendedly dominated” by dostarlimab (higher cost and lower effectiveness), meaning it was not on the efficient frontier (Dioun et al., 2023). The relevant comparison was thus dostarlimab vs PLD: here, dostarlimab added substantial costs (total ∼$151,533 per patient) for the QALY gain achieved, resulting in an ICER of $331,913 per QALY (Dioun et al., 2023). At a $100,000/QALY threshold, this is well above acceptable. One-way sensitivity analyses indicated that a ∼ 52 % reduction in dostarlimab’s price (to approximately $4,900 per dose) would be required for it to become cost-effective in this population. In probabilistic analysis, the likelihood of dostarlimab being cost-effective at $100 K/QALY was essentially zero. Therefore, although dostarlimab greatly improves survival outcomes in dMMR recurrent endometrial cancer (providing what the authors describe as “greater survival” than other treatments) (Dioun et al., 2023), its current cost is prohibitive from a cost-effectiveness standpoint under typical U.S. thresholds. These findings echo the broader theme: immunotherapy for endometrial cancer delivers meaningful health benefits but needs significant price reductions or value-based pricing strategies to align with conventional definitions of “cost-effective” care.

## 4 Conclusion

The cost-effectiveness analyses reviewed indicate that while new immunotherapy-based combinations for advanced endometrial cancer can provide substantial clinical benefits, their economic value is highly variable across settings and patient subgroups. In general, treatments targeting dMMR/MSI-H tumors – which confer the greatest survival gains – are much more likely to be cost-effective than those for pMMR disease, given current drug prices. First-line ICI + chemotherapy regimens appear to deliver good value in dMMR patients under U.S. willingness-to-pay thresholds (approximately $150,000 per QALY), with ICERs well below that benchmark in multiple analyses (Huo et al., 2024, Huo et al., 2024). However, the same regimens in pMMR patients yield far higher ICERs, often exceeding typical thresholds (Huo et al., 2024, Huo et al., 2024, Liu et al., 2025). In second-line therapy for pMMR cancers, the ICERs for pembrolizumab + lenvatinib were above accepted cutoffs in both U.S. and Chinese analyses (Diggs et al., 2023, Sun et al., 2022), reflecting the modest incremental survival in this context. Conversely, for recurrent dMMR cancers, immunotherapy monotherapy (dostarlimab or pembrolizumab) improves outcomes but remains too costly to be considered cost-effective at standard thresholds (Dioun et al., 2023) without significant price reductions.

These studies underscore the importance of drug pricing and patient selection in determining value. Many authors found that relatively small price discounts (on the order of 5–15 %) could change a regimen from not cost-effective to cost-effective (Kim et al., 2023, Diggs et al., 2023). Others noted that using immunotherapy primarily in the biomarker-defined subgroup most likely to benefit (dMMR patients) maximizes cost-effectiveness (Huo et al., 2024, Liu et al., 2025). Health systems may consider negotiating lower prices for these agents or implementing value-based pricing schemes to improve the cost per QALY. As additional data emerge – for example, longer-term survival outcomes, real-world utility values, or the entry of biosimilars/generics – the cost-effectiveness of these regimens should be reappraised.

In conclusion, integrating ICIs into therapy for advanced endometrial cancer offers clear clinical advantages, particularly for dMMR tumors, but policymakers and clinicians must weigh the substantial costs. At current prices, pembrolizumab or dostarlimab combined with chemotherapy is likely an efficient use of resources for advanced **dMMR** endometrial cancer, whereas their use in **pMMR** disease or in later-line settings may not be economically justified unless drug costs decrease. Careful consideration of cost-effectiveness evidence – alongside efficacy and safety – can help guide optimal use of these novel therapies to ensure both improved patient outcomes and sustainable healthcare spending.

**Declaration of generative AI and AI-assisted technologies in the writing process**

During the preparation of this work, the author used ChatGPT (OpenAI) in order to assist with English language refinement and organization of literature summaries. After using this tool, the author reviewed and edited the content as needed and takes full responsibility for the content of the publication.

## CRediT authorship contribution statement

**Katsuaki Inami:** Writing – review & editing, Writing – original draft, Visualization, Project administration, Methodology, Investigation, Formal analysis, Data curation, Conceptualization.

## Funding

No external funding was received for this study.

## Declaration of competing interest

The authors declare that they have no known competing financial interests or personal relationships that could have appeared to influence the work reported in this paper.
